# Synergistic impact of dysglycemia and HPV on cervical cancer risk: a potential mediating role of Ki-67

**DOI:** 10.3389/fendo.2025.1422881

**Published:** 2025-10-06

**Authors:** Yulong Zhang, Junxin Zhang, Haibo Li, YiLing Zhuang, Qianru You, Yanzhao Su, Xiangqin Zheng, Suyu Li

**Affiliations:** ^1^ Department of Gynecology, Fujian Province Key Clinical Specialty for Gynecology, National Key Gynecology Clinical Specialty Building Institution, Fujian Maternity and Child Health Hospital, College of Clinical Medical for Obstetrics & Gynecology and Pediatrics, Fujian Medical University, Fuzhou, China; ^2^ Fujian Maternity and Child Health Hospital, College of Clinical Medicine for Obstetrics & Gynecology and Pediatrics, Fujian Medical University, Fuzhou, China

**Keywords:** Dysglycemia, HPV, Ki-67, cervical cancer, Interaction OR00: OR(HPV: negative, Hyperglycemia: negative), OR01: OR(HPV: Positive Hyperglycemia: negative), OR10: OR(HPV: negative, Hyperglycemia: positive), OR11: OR(HPV: positive, Hyperglycemia: positive)

## Abstract

**Background:**

Cervical cancer, linked to HPV and dysglycemia, lacks clarity on their combined impact. This study explores Ki-67’s role in mediating HPV and dysglycemia effects on cervical cancer risk.

**Methods:**

This study enrolled patients with abnormal cervical cancer screening results, undergoing colposcopy and conization at Fujian Maternity and Child Health Hospital’s Cervical Disease Center from June 2018 to June 2023. Statistical analyses compared baseline characteristics across cervical lesion categories. Multinomial logistic regression examined HPV and dysglycemia associations with LSIL (low-grade squamous intraepithelial lesions), HSIL(high-grade squamous intraepithelial lesions), and cervical cancer, highlighting interaction and mediation analyses involving Ki-67.

**Results:**

A total of 4,115 participants were included: 573 with hyperglycemia, 1,479 with HPV only, and 548 with both HPV and hyperglycemia. Prediabetes and diabetes significantly increased cancer risk (OR: 2.47, 95% CI: 1.75-3.47 and OR: 3.67, 95% CI: 2.41-5.6, respectively). Coexisting hyperglycemia further elevated cervical cancer risk by over three-fold (OR: 3.12, 95% CI: 2.34-4.16) compared to HPV-positive normoglycemics. A significant interaction between hyperglycemia and HPV infection was observed (AP (attributable proportion): 0.69, 95% CI: 0.61-0.77, p<0.001; SI (synergy index): 3.27, 95% CI: 2.5-4.27, p<0.001). Ki-67+ expression accounted for 39.84%, 37.35%, and 55.18% of the total effect of hyperglycemia, HPV, and their combined impact, respectively. Additionally, the combination of dysglycemia and HPV had a significant indirect effect on Ki-67 levels (estimate: 0.08, 95% CI: 0.06- 0.09, p<0.001).

**Conclusions:**

Dysglycemia and HPV infection synergistically elevate cervical cancer risk, possibly influenced by Ki-67. Effective screening and management for both are vital in prevention. Further research is required to validate findings and elucidate molecular mechanisms.

## 1 Introduction

Cervical cancer stands as a major global health challenge, ranking as the second most prevalent cancer among women worldwide, resulting in over 250,000 deaths annually (1), with an estimated 604,000 new cases and 342,000 deaths reported in 2020 (2). Human papillomaviruses (HPV16 and HPV18) have been identified as potent catalysts in the development of cervical cancer in women (3), underscoring the critical importance of understanding and addressing HPV infection. However, the landscape of cervical cancer risk is not solely defined by viral factors. Diabetes, characterized by metabolic dysregulation and immune alterations, emerges as a significant contributor to the burden of cervical cancer (4, 5).

Contrary to being a mere bystander in cervical carcinogenesis, diabetes is increasingly recognized for its intricate involvement in driving disease progression. The global epidemic of diabetes further compounds the cervical cancer burden, with the condition affecting over 8% of adults worldwide (6). In China alone, recent data from 2018 reveal a notable surge in diabetes prevalence, with rates soaring to 12.4%. Moreover, antecedent diabetes is observed in 38.1% of cases, while diabetes and prediabetes together afflict 50.5% of the population. Strikingly, nearly half of the affected individuals are women, with a weighted percentage of 49.5% (7).

Investigating the intricate relationship between diabetes and cancer is an area of active research, with diverse findings and ongoing debate (8–18). Some studies suggest that diabetes escalates cervical cancer risk (8–10) 8-10, and others also indicate that metformin, a commonly prescribed medication for diabetes, may potentially mitigate this risk (19–21), particularly with cumulative use exceeding 2 years (21). In a cross-sectional study involving 791 women, it was found that diabetes and prediabetes are associated with cervical cancer in patients with high-risk HPV (HR-HPV) combined with high-grade squamous intraepithelial lesions (HGSIL) (22).

Given the substantial global burden of cervical cancer, particularly in developing nations, and the emerging evidence linking diabetes to an increased risk of cervical cancer development and progression, further research is crucial to elucidate the underlying mechanisms and develop effective strategies for prevention, early detection, and management of these intertwined health challenges.

Several studies have highlighted the link between dysglycemia and cervical neoplasia (8–10, 22), yet the potential synergistic effect of dysglycemia on HPV-driven cervical cancer progression remains unexplored. Furthermore, the underlying mechanisms through which dysglycemia may potentiate HPV-mediated cervical carcinogenesis are still elusive. Remarkably, the proliferation marker Ki-67 has been implicated as a crucial player in HPV-driven cervical cancer progression (23). Notably, Ki-67 staining offers improved specificity for triaging HPV-positive women with cervical precancerous lesions (24). Interestingly, Ki-67 has also been found to contribute to the promotion of other malignancies in the context of dysglycemia (25, 26). This intriguing observation raises an important question: Could the potential synergistic effect of dysglycemia on HPV-induced cervical cancer progression be mediated through a mechanism involving Ki-67?

Given the global cervical cancer burden and rising metabolic disorders, exploring dysglycemia’s role alongside HPV in cervical carcinogenesis is crucial. Understanding dysglycemia’s role in HPV-driven cancer and Ki-67’s involvement could inform novel therapies and personalized management. This sheds light on biomarker potential for early detection and risk assessment. Hence, conducting comprehensive research is crucial to investigate the potential synergistic impact of dysglycemia on HPV-driven cervical cancer progression, along with the mechanistic role of Ki-67 in this complex process. Therefore, this study delved into the intricate interplay of Ki-67 in mediating the effects of Human Papillomavirus (HPV) infection and dysglycemia on cervical cancer risk.

## 2 Materials and methods

### 2.1 Study population

**Figure 1:**
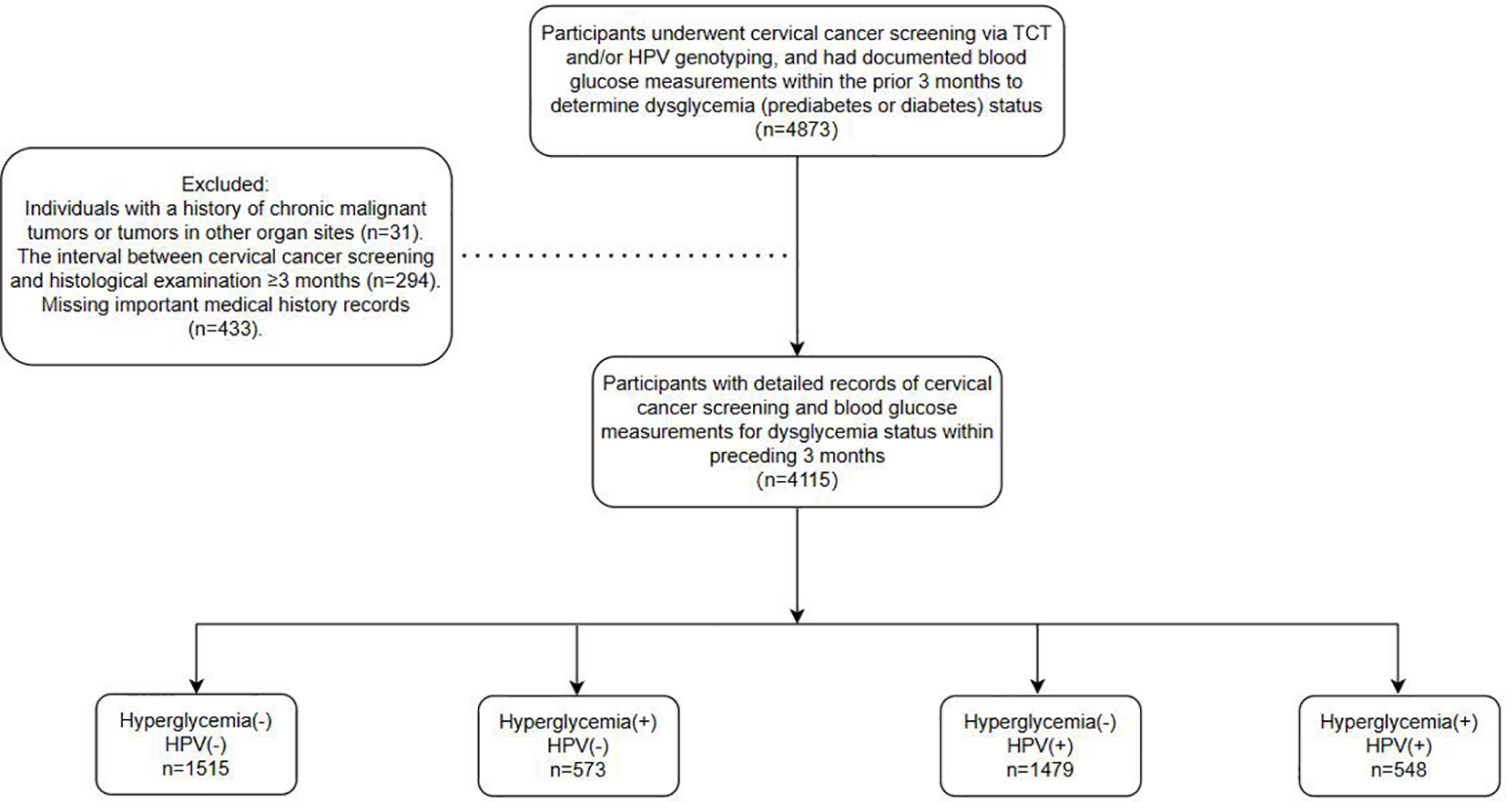
Flowchart of patient enrollment.

This observational study recruited participants from patients referred to the Cervical Disease Center of Fujian Maternity and Child Health Hospital for colposcopy and conization procedures following abnormal cervical cancer screening results between June 2018 and June 2023. Screening methods employed included the ThinPrep Cytology Test (TCT) and/or HPV genotyping, with a maximum interval of 3 months between screening and histological examination. Furthermore, the study encompassed a total of 4,155 patients. All participants had undergone blood glucose testing within the preceding 3 months, with documented results available. Dysglycemia, including both prediabetes and diabetes, was defined according to the American Diabetes Association (ADA) criteria using SI units: prediabetes as fasting plasma glucose (FPG) between 5.6–6.9 mmol/L and/or HbA1c between 5.7%–6.4%; and diabetes as FPG ≥7.0 mmol/L and/or HbA1c ≥6.5%. Participants with either abnormal FPG or HbA1c were classified as having dysglycemia. Clinical parameters such as age, gravidity, parity, blood glucose profiles (fasting and postprandial levels, and glycosylated hemoglobin), BMI, HPV genotypes, and cervical pathology were obtained from medical records. Ultimately, the study encompassed a cohort of 4155 patients, with the detailed workflow depicted in **Figure 1**.

Ethical approval was obtained from the Ethics Committee of Fujian Maternity and Child Health Hospital, Affiliated Hospital of Fujian Medical University (2023KY038), in accordance with the Declaration of Helsinki (2013 revision). Due to the retrospective design, informed consent was waived.

### 2.2 Serologic detection and HPV genotyping

Fasting and postprandial blood glucose levels were measured to evaluate participants’ glucose metabolism status, providing insights into their glycemic control both in the fasting state and after meals. These measurements help identify individuals with impaired glucose tolerance or diabetes mellitus, important risk factors for cervical cancer. Glycosylated hemoglobin (HbA1c) levels were quantified to assess participants’ long-term glucose control. HbA1c reflects average blood glucose levels over the past 2–3 months and is a valuable indicator of overall glycemic management. Lipid metabolism-related parameters, including cholesterol, triglycerides, and low-density lipoprotein cholesterol (LDL-C) levels, were also evaluated. Dyslipidemia, characterized by abnormal lipid profiles, has been associated with an increased risk of various cancers, including cervical cancer. Assessing lipid parameters provides additional insights into participants’ metabolic health and potential cancer risk.

Moreover, HPV detection using polymerase chain reaction-reverse dot blot (PCR-RDB) technology was performed to identify specific HPV genotypes present in cervical samples. HPV genotyping utilized the PCR-RDB HPV genotyping method provided by Yaneng Biotech, distinguishing among 18 types of high-risk HPV (HR-HPV) strains (16, 18, 31, 33, 35, 39, 45, 51, 52, 53, 56, 58, 59, 66, 68, 73, 82, and 83). Single identification of a high-risk HPV type indicated positivity, while detection of multiple HPV types in a sample suggested multiple infections.

### 2.3 Pathological diagnosis and Ki-67 staining in cervical biopsy samples

Colposcopy and cervical biopsy were performed in all patients. Colposcopy and cervical biopsy are performed by qualified attending physicians and above in the Cervical Center of our hospital. Biopsy was performed from the abnormal area using a perforated cervical biopsy tissue forceps. Cervical specimens were processed in a histopathology laboratory, where histopathologists performed a blind diagnosis of HPV status in participants. Two blind senior pathologists independently performed pathological assessments of cervical biopsy, ECC and cone tissue. Histological end points were defined according to WHO Classification of Female Genital Tumors (4th Edition) 2014, and were divided into normal or cervicitis, low-grade cervical intraepithelial neoplasia(LSIL), high-grade cervical intraepithelial neoplasia(HSIL), and cervical cancer.

Ki-67 staining kit was used for immunocytochemical staining of each biopsy tissue specimen. The scoring criteria for the Ki-67 index involve assessing the percentage of positive cells. Typically, an appropriate field of view is selected, and the proportion of Ki-67 positive cells within that field is calculated. This proportion is usually expressed as a percentage. To ensure accuracy, multiple fields are often chosen for scoring, and the results are averaged. Following scoring, the results are verified and recorded. For each sample, the percentage of Ki-67 positive cells is documented as a quantitative measure of the Ki-67 index. For the purpose of this study, high Ki-67 expression was defined as ≥10% positive cells, based on established literature and clinical relevance. The scoring process requires highly trained professionals to ensure accuracy and reliability. Three pathologists typically independently evaluate the Ki-67 index to minimize subjective bias and ensure result consistency.

### 2.4 Statistical analysis

Participant characteristics were presented as mean ± SD for continuous variables and as frequency and percentage for categorical variables. Baseline characteristics across different categories of cervical lesions were compared using one-way analysis of variance (ANOVA) or the Kruskal-Wallis rank-sum test for continuous variables, and the Chi-squared test or Fisher’s exact test for categorical variables. Multinomial logistic regression was conducted to investigate the associations of HPV and dysglycemia, both separately and in combination, with the occurrence of LSIL, HSIL, and cervical cancer. Adjusted odds ratios (ORs) with 95% confidence intervals (CIs) were calculated, and trend analysis was performed. Interaction analysis was also employed to assess multiplicative scale, relative excess risk due to interaction (RERI), attribute proportion (AP), and synergy index (SI) for various cervical lesions. Additionally, mediation analysis was conducted to examine whether the combined co-infection of HPV and dysglycemia influences LSIL, HSIL, and cervical cancer through Ki-67 expression. The mediation proportion was estimated using the R package “mediation”, with a 95% confidence interval calculated around the estimate. Mediation diagrams were also generated to illustrate these relationships. Statistical significance was defined as a two-sided test with a significance level of P ≤ 0.05. All statistical analyses were performed using R, version 4.2.2 (http://www.R-project.org).

## 3 Results

### 3.1 The impact of blood glucose levels on HPV infection and cervical lesions

**Table 1** provides data on 4,115 individuals, investigating the interplay between HPV infection, cervical lesion severity, and various clinical parameters, notably blood glucose levels. HPV prevalence showed significant associations with cervical lesion severity (p < 0.001). Among the total cohort, 49.3% tested positive for HPV, rising sharply to 98.9% in the cervical cancer group. Similarly, high-risk HPV types 16/18 prevalence increased from 16.7% overall to 65% in the cancer group (p < 0.001). Multiple HPV infections were more prevalent in higher cervical lesion grades (p < 0.001). Dysglycemia emerged as a crucial risk factor. While only 8.4% had diagnosed diabetes, 18.9% exhibited prediabetes based on elevated blood glucose levels. The mean fasting blood glucose was 5.3 mmol/L overall but elevated to 5.9 mmol/L in the cancer group (p < 0.001). Postprandial 2-hour glucose levels were also significantly higher in the cancer group at 8.0 mmol/L compared to 7.0 mmol/L overall (p < 0.001). HbA1c, reflecting longer-term glycemic control, similarly increased with lesion severity from 6.3% to 7.1% in cancer (p < 0.001).

**Table 1:** Basic characteristics and the impact of blood glucose levels on HPV infection and cervical lesions.

Variables	Total (n = 4115)	None (n = 1515)	Hyperglycemia (n = 573)	ONLY HPV (n = 1479)	HPV and Hyperglycemia (n = 548)	p	Statistic
Age, Mean ± SD	41.6 ± 11.7	40.9 ± 10.3	46.9 ± 12.4	38.8 ± 10.8	46.0 ± 13.4	< 0.001	103.108
Parity, Mean ± SD	1.5 ± 1.3	1.5 ± 1.2	1.7 ± 1.3	1.4 ± 1.3	1.8 ± 1.5	< 0.001	16.248
Gravidity, Mean ± SD	2.3 ± 2.2	2.3 ± 2.2	2.6 ± 2.1	2.2 ± 2.1	2.6 ± 2.2	< 0.001	8.148
Fasting Blood Sugar, Mean ± SD	5.3 ± 1.2	4.8 ± 0.5	6.5 ± 1.7	4.8 ± 0.5	6.6 ± 1.6	< 0.001	915.385
Diabetes classification, n (%)						< 0.001	4116.332
None	2994 (72.8)	1515 (100)	0 (0)	1479 (100)	0 (0)		
Diabetes	345 (8.4)	0 (0)	181 (31.6)	0 (0)	164 (29.9)		
Pre-diabetes	776 (18.9)	0 (0)	392 (68.4)	0 (0)	384 (70.1)		
Glycated Hemoglobin, Mean ± SD	6.3 ± 1.3	5.4 ± 0.3	6.6 ± 1.2	5.4 ± 0.3	6.7 ± 1.5	< 0.001	43.102
BMI Index, Mean ± SD	26.6 ± 11.6	25.8 ± 3.1	30.3 ± 31.3	25.9 ± 3.1	26.8 ± 3.2	0.03	2.992
Postprandial 2h, Mean ± SD	7.0 ± 2.9	5.6 ± 1.0	8.6 ± 3.5	5.6 ± 1.0	8.5 ± 3.5	< 0.001	101.058
Number of Infections, Mean ± SD	0.7 ± 0.9	0.0 ± 0.0	0.0 ± 0.0	1.4 ± 0.8	1.4 ± 0.8	< 0.001	2020.592
Multiple infection classification, n (%)						< 0.001	4115.279
None	2088 (50.7)	1515 (100)	573 (100)	0 (0)	0 (0)		
Single	1499 (36.4)	0 (0)	0 (0)	1097 (74.2)	402 (73.4)		
Multiple	528 (12.8)	0 (0)	0 (0)	382 (25.8)	146 (26.6)		
Cervical lesion classification, n (%)						< 0.001	739.185
None	2796 (67.9)	1237 (81.7)	486 (84.8)	831 (56.2)	242 (44.2)		
HSIL	280 (6.8)	30 (2)	9 (1.6)	181 (12.2)	60 (10.9)		
LSIL	762 (18.5)	246 (16.2)	77 (13.4)	330 (22.3)	109 (19.9)		
Cancer	277 (6.7)	2 (0.1)	1 (0.2)	137 (9.3)	137 (25)		
Ki-67, Mean ± SD	8.0 ± 15.3	3.8 ± 9.4	3.5 ± 9.2	10.9 ± 17.9	16.6 ± 19.9	< 0.001	143.691
TC, mmol/L, Mean ± SD	5.2 ± 1.2	5.2 ± 1.2	5.1 ± 1.2	5.2 ± 1.2	5.3 ± 1.2	0.198	1.556
H-DLC, mmol/L, Mean ± SD	1.5 ± 0.4	1.5 ± 0.4	1.4 ± 0.4	1.5 ± 0.4	1.5 ± 0.4	< 0.001	24.422
L-DLC, mmol/L, Mean ± SD	2.9 ± 0.9	2.9 ± 0.9	2.9 ± 0.9	2.9 ± 0.9	2.9 ± 0.9	0.25	1.371
TG,mmol/L, Mean ± SD	1.8 ± 1.3	1.7 ± 1.2	2.0 ± 1.6	1.7 ± 1.3	2.0 ± 1.4	< 0.001	17.813
Albumin.g/L, Mean ± SD	40.0 ± 4.7	40.2 ± 4.5	39.6 ± 4.8	40.2 ± 4.7	39.7 ± 4.8	0.015	3.472
AST, U/L, Mean ± SD	18.6 ± 16.6	18.1 ± 16.0	21.4 ± 18.7	17.7 ± 16.5	19.7 ± 15.8	< 0.001	8.236
ALT, U/L, Mean ± SD	19.2 ± 10.2	19.1 ± 10.3	20.3 ± 12.2	18.6 ± 8.9	19.8 ± 10.9	0.005	4.334
γ-GT, U/L, Mean ± SD	20.9 ± 37.8	19.0 ± 23.0	30.5 ± 87.7	18.2 ± 14.3	23.2 ± 22.6	< 0.001	16.234
TBIL,μmol/L, Mean ± SD	9.6 ± 4.7	9.8 ± 4.7	9.6 ± 4.6	9.4 ± 4.4	9.8 ± 5.5	0.226	1.451
TP, g/L, Mean ± SD	67.8 ± 6.7	67.8 ± 6.5	67.2 ± 7.3	68.2 ± 6.7	67.4 ± 6.7	0.009	3.896

This table presents baseline clinical and metabolic characteristics of the total study population (n = 4,115), stratified by HPV and blood glucose status:

None: HPV-negative and normoglycemic.

Hyperglycemia only: HPV-negative and hyperglycemic.

HPV only: HPV-positive and normoglycemic.

HPV + Hyperglycemia: Coexistence of HPV infection and hyperglycemia.

Continuous variables are expressed as mean ± standard deviation and analyzed using one-way ANOVA. Categorical variables are expressed as counts and percentages, analyzed using the Chi-square test.

TC, total cholesterol; H-DLC, High-density Lipoprotein Cholesterol; L-DLC, Low-density Lipoprotein Cholesterol; TG, triglyceride; AST, aspartate aminotransferase; ALT, Alanine Aminotransferase; γ-GT, γ-glutamyl transpeptidase; TBIL, total bilirubin; TP, total protein; HPV, Human Papillomavirus; HBV, Hepatitis B Virus.

Additionally, focusing solely on the HPV-positive subgroups, individuals with concurrent dysglycemia exhibited a significantly higher prevalence of cervical cancer at 25% compared to 9.3% in the only HPV-positive group (p < 0.001). The proliferative marker Ki-67 was also notably elevated at 16.6% in the dysglycemic HPV-positive subgroup. Diabetes classification demonstrated a substantial association with both HPV infection and cervical lesions (p < 0.001), indicating elevated rates of diabetes and prediabetes among individuals with advanced lesions, suggesting an association between dysglycemia and HPV-driven cervical carcinogenesis. Other metabolic parameters such as dyslipidemia and liver enzyme elevations were similarly linked to more severe cervical lesions, potentially reflecting shared metabolic dysfunction. However, BMI did not exhibit significant differences between groups.

In summary, these findings underscore that hyperglycemia and diabetes pose significant risks for HPV persistence and progression to precancerous cervical lesions and cancer. Tight glycemic control may confer protection, as evidenced by the increasing levels of fasting glucose, postprandial glucose, and HbA1c across worsening cervical pathology grades. This emphasizes the importance of metabolic health in HPV immunity and cervical cancer prevention efforts.

### 3.2 Risk association between diabetes/prediabetes and HPV-driven cervical lesion progression

**Table 2** explores the association between blood glucose levels, HPV infection, and cervical lesions. The data examines the relationship between dysglycemia (diabetes and prediabetes), HPV infection status, HPV genotypes, and severity of cervical lesions in 4,115 individuals. Examining HPV genotypes, infection with high-risk HPV markedly increased odds of cervical cancer by over 152-fold compared to HPV negative individuals (OR: 152.85, 95% CI: 48.56–481.09, p<0.001). Among those with dysglycemia, 10.3% had cervical cancer compared to only 4.6% in the normoglycemic group (p<0.001). Prediabetes and diabetes were both significantly associated with increased cancer risk(OR: 2.47, 95% CI: 1.75-3.47 and OR:3.67, 95% CI: 2.41-5.6, respectively). HPV prevalence was significantly higher in the dysglycemic group (p<0.001). When stratified by HPV and dysglycemia status, those with both HPV and dysglycemia had the highest cervical cancer rate at 25% versus 9.3% with HPV alone and 0.1% with neither (p<0.001). The combination of high-risk HPV and dysglycemia appeared to promote cervical lesion progression.

**Table 2:** Risk association between diabetes/prediabetes and HPV-driven cervical lesion progression.

Variables	n.Total	n.Ref_%	n.LSIL_%	n.HSIL_%	n.CANCER_%	LSIL.vs.Ref_OR.95CI	P.value_1	HSIL.vs.Ref_OR.95CI	P.value_2	CANCER.vs.Ref_OR.95CI	P.value_3
HPV Typing											
Hpv-negative	2088	1723 (82.5)	323 (15.5)	39 (1.9)	3 (0.1)	1(Ref)		1(Ref)		1(Ref)	
Hpv-positive	2027	1073 (52.9)	439 (21.7)	241 (11.9)	274 (13.5)	2.14 (1.81– 2.52)	<0.001	10.18 (7.19–14.41)	<0.001	152.85 (48.56– 481.09)	<0.001
Abnormal blood sugar											
None	2994	2068 (69.1)	576 (19.2)	211 (7)	139 (4.6)	1(Ref)		1(Ref)		1(Ref)	
Pre-diabetes	776	499 (64.3)	146 (18.8)	51 (6.6)	80 (10.3)	–	0.214	–	0.923	2.47 (1.75– 3.47)	<0.001
Diabetes	345	229 (66.4)	40 (11.6)	18 (5.2)	58 (16.8)	–	0.093	–	0.354	3.67 (2.41– 5.6)	<0.001
HPV and Hyperglycemia											
None	1515	1237 (81.7)	246 (16.2)	30 (2)	2 (0.1)	1(Ref)		1(Ref)		1(Ref)	
Hyperglycemia	573	486 (84.8)	77 (13.4)	9 (1.6)	1 (0.2)	–	0.332	–	0.363	–	0.998
ONLY HPV	1479	831 (56.2)	330 (22.3)	181 (12.2)	137 (9.3)	1.92 (1.59– 2.33)	<0.001	9.22 (6.19–13.73)	<0.001	107.47 (26.39– 437.59)	<0.001
HPV and Hyperglycemia	548	242 (44.2)	109 (19.9)	60 (10.9)	137 (25)	2.29 (1.76– 3)	<0.001	9.83 (6.19–15.61)	<0.001	337.73 (82.57– 1381.35)	<0.001

This table presents the relationship between HPV status and blood glucose abnormalities (prediabetes and diabetes) with the progression of cervical lesions, categorized into normal (reference), LSIL, HSIL, and cancer. Multinomial logistic regression was used to calculate odds ratios (ORs) and 95% confidence intervals (CIs), comparing LSIL, HSIL, and cancer groups to the reference group. *To avoid confusion, only statistically significant associations are interpreted in the main text. Non-significant estimates are indicated by “–.”*

LSIL, Low-grade Squamous Intraepithelial Lesion; HSIL, High-grade Squamous Intraepithelial Lesion; OR, Odds Ratio; HPV, Human Papillomavirus.

### 3.3 Subgroup analysis: combined effects of HPV infection and hyperglycemia on cervical lesions

**Table 3** stratifies individuals into subgroups based on HPV status and presence of hyperglycemia (diabetes/prediabetes). This allows examination of how hyperglycemia impacts cervical lesion risk in both HPV-positive and HPV-negative settings. Among the HPV-negative subgroups, hyperglycemia alone did not significantly increase risk of low-grade squamous intraepithelial lesions (LSIL) or higher grade cervical lesions compared to normoglycemic controls. However, in the HPV-positive setting, coexisting hyperglycemia drastically elevated cervical cancer risk over 3-fold (OR: 3.12, 95% CI: 2.34-4.16) versus HPV-positive normoglycemics. 25% of the HPV-positive hyperglycemic subgroup had invasive cancer compared to only 9.3% with HPV alone.

**Table 3:** Subgroup analysis: combined effects of HPV infection and hyperglycemia on cervical lesions.

Subgroup		n.Total	n.Ref_%	n.LSIL_%	n.HSIL_%	n.CANCER_%	LSIL.vs.Ref_OR.95CI	P.value_1	HSIL.vs.Ref_OR.95CI	P.value_2	CANCER.vs.Ref_OR.95CI	P.value_3
Hpv-negative												
	Normal	1515	1237 (81.7)	246 (16.2)	30 (2)	2 (0.1)	1(Ref)		1(Ref)		1(Ref)	
	Hyperglycemia	573	486 (84.8)	77 (13.4)	9 (1.6)	1 (0.2)	–	0.107	–	0.482	–	0,718
Hpv-positive												
	Normal	1479	831 (56.2)	330 (22.3)	181 (12.2)	137 (9.3)	1(Ref)		1(Ref)		1(Ref)	
	Hyperglycemia	548	242 (44.2)	109 (19.9)	60 (10.9)	137 (25)	–	0.342	–	0.435	3.12 (2.34–4.16)	<0.001

This table shows the relationship between hyperglycemia and cervical lesion progression in subgroups stratified by HPV infection status. Multinomial logistic regression was used to calculate odds ratios (ORs) and 95% confidence intervals (CIs) for LSIL, HSIL, and cervical cancer versus normal pathology, using HPV-negative normoglycemic women as the reference group for each stratum. *To avoid confusion, only statistically significant associations are interpreted in the main text. Non-significant estimates are indicated by “–.”*

LSIL, Low-grade Squamous Intraepithelial Lesion; HSIL, High-grade Squamous Intraepithelial Lesion; OR, Odds Ratio; HPV, Human Papillomavirus.

### 3.4 Additive and multiplicative interaction between dysglycemia and HPV infection on cervical cancer risk

**Figure 2:**
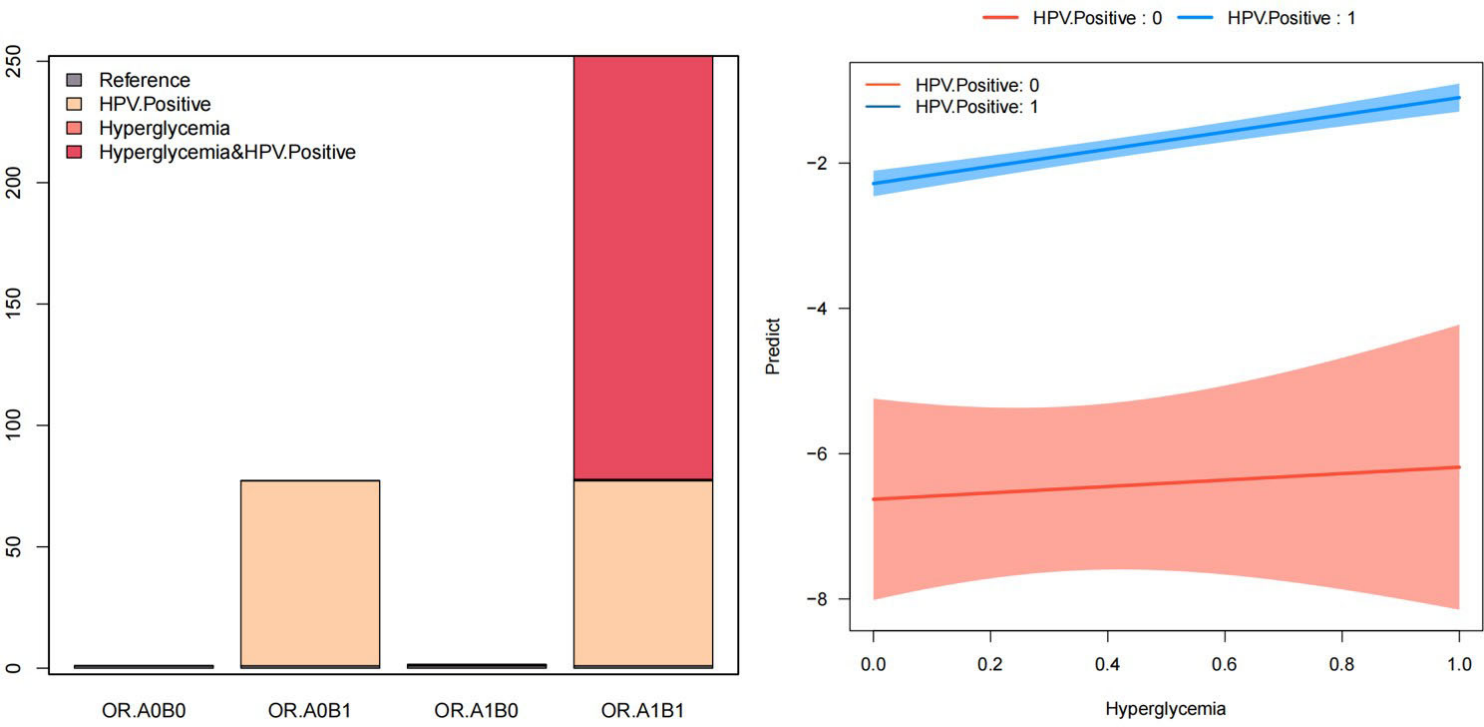
Interaction analysis: dysglycemia and HPV infection.

**Table 4** and **Figure 2** depict the additive and multiplicative interactions between dysglycemia and HPV infection in relation to cervical cancer risk. The OR for women who are HPV positive and have hyperglycemia (OR11) is 252.17 (95% CI: 62.17-1022.81, p < 0.001), indicating a significantly increased risk compared to those without either exposure. Being HPV positive alone (OR01) also substantially increases the odds of cervical cancer by 77.23 times (95% CI: 19.08-312.52, p < 0.001). In contrast, hyperglycemia alone (OR10) does not appear to significantly elevate the risk, and therefore, was excluded from further interpretation.

**Table 4:** Additive and multiplicative interaction between dysglycemia and HPV infection on cervical cancer risk.

Measures	Estimates	CI.95.low	CI.95.up	P.value
OR00	1			
OR01	77.23	19.08	312.52	<0.001
OR10	–	–	–	0.72
OR11	252.17	62.17	1022.81	<0.001
OR(HPV.Positive on outcome [Hyperglycemia==0]	77.23	19.08	312.52	<0.001
OR(HPV.Positive on outcome [Hyperglycemia==1]	162	22.56	1163.37	<0.001
OR(Hyperglycemia on outcome [HPV.Positive==0]	–	–	–	0.72
OR(Hyperglycemia on outcome [HPV.Positive==1]	3.27	2.51	4.24	<0.001
Multiplicative scale	–	–	–	0.55
RERI	–	–	–	0.08
AP	0.69	0.61	0.77	<0.001
SI	3.27	2.5	4.27	<0.001

This table summarizes the joint and individual effects of HPV infection and hyperglycemia on cervical cancer risk, as well as their interaction on both additive and multiplicative scales. Only statistically significant estimates (p < 0.05) are interpreted. Odds ratios (ORs) were calculated using logistic regression models; interaction measures were derived using standard epidemiological formulas; to avoid confusion, only statistically significant associations are interpreted in the main text. Non-significant estimates are indicated by “–.”

Model adjusted for age, pregnancy, parity, total cholesterol, triglyceride, High -density lipoprotein cholesterol, Low density lipoprotein cholesterol, Albumin, Alanine aminotransferase, γ-glutamyl transpeptidase, total bilirubin, Specific protein.

OR00: OR(HPV: negative, Hyperglycemia: negative);

OR01: OR(HPV: Positive, Hyperglycemia: negative);

OR10: OR(HPV: negative, Hyperglycemia: positive);

OR11: OR(HPV: positive, Hyperglycemia: positive);

Relative excess risk due to interaction:RERI = ORA1B1 - ORA0B1 - ORA1B0 + 1;2.attribute proportion:AP = RERI/A1B1;3. synergy index:SI = (ORA1B1 - 1)/[(ORA0B1 - 1) + (ORA1B0 - 1)];4.Multiplicative scale (P value) ||| In the absence of interaction AP = 0 and RERI and S = 1.”

RERI, Relative Excess Risk due to Interaction; AP, Attributable Proportion due to Interaction; SI, Synergy Index.

**Table 4** also provides evidence of a potential interaction between these two factors on cervical cancer risk. The multiplicative scale suggests a multiplicative interaction, though it is not statistically significant. Additionally, the attributable proportion due to interaction (AP) of 0.69 (95% CI: 0.61-0.77, p < 0.001) indicates that 69% of the combined effect is attributable to the interaction between HPV and hyperglycemia. Furthermore, the synergy index (SI) of 3.27 (95% CI: 2.5-4.27, p < 0.001) demonstrates a synergistic effect of these two exposures on cervical cancer risk.

### 3.5 Role of Ki-67 in mediating the interaction of HPV infection and dysglycemia in cervical cancer

**Figure 3:**
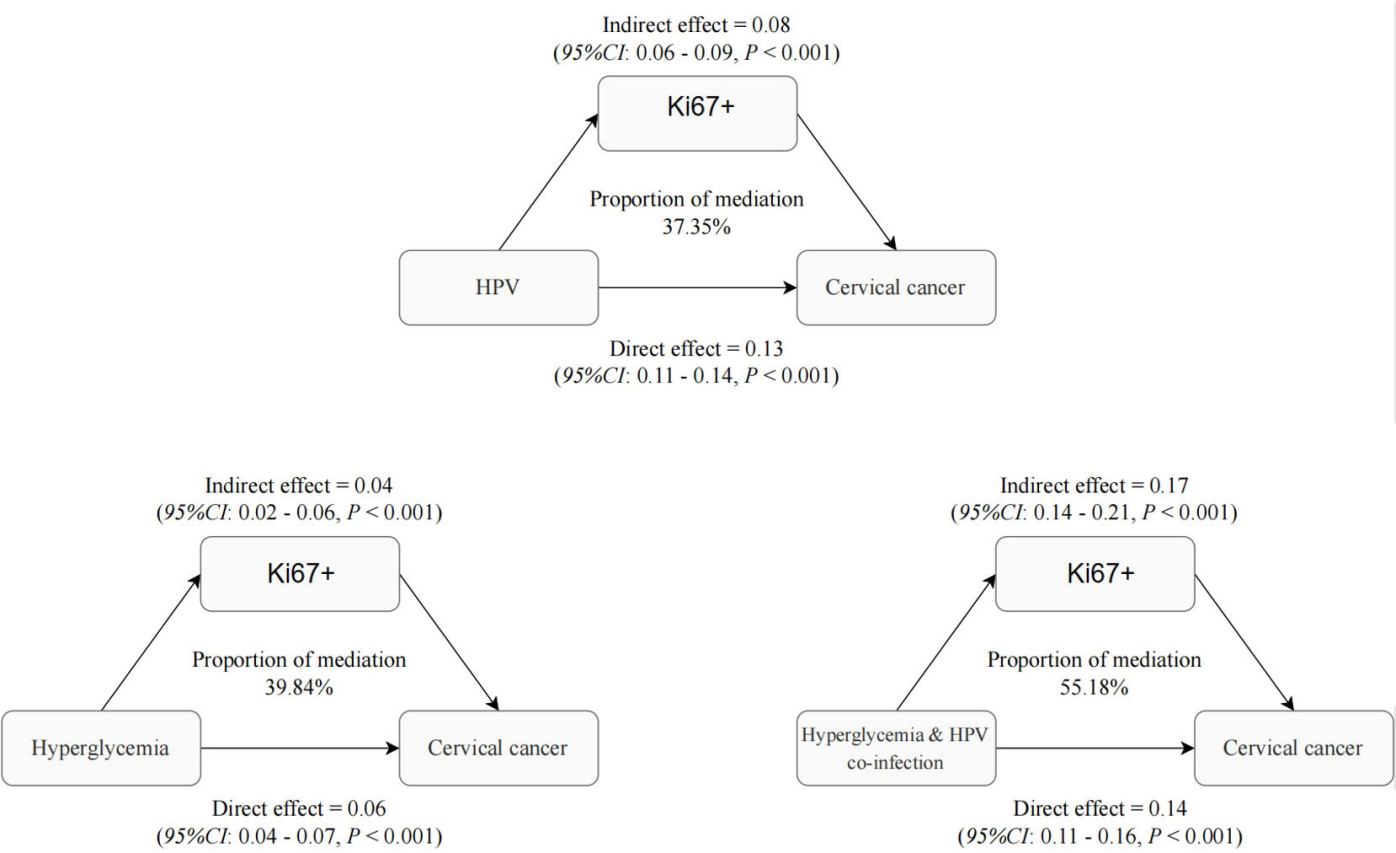
Role of Ki-67 in mediating the interaction of HPV infection and dysglycemia in cervical cancer.

**Figure 3** predominantly focuses on the mediation effects of Hyperglycemia, HPV, and their combined impact on cervical cancer through the Ki-67 pathway. Ki-67, a protein associated with cellular proliferation, serves as a critical marker for assessing cancer cell growth and division rates. The mediation analysis conducted in the study yielded significant insights into the role of Ki-67+ in mediating the effects of Hyperglycemia, HPV, and their combined impact on cervical cancer. The results indicated that Ki-67+ accounted for a substantial proportion of the total effect of Hyperglycemia, HPV, and their co-infection on these cervical abnormalities. Specifically, the analysis revealed that Ki-67+ accounted for 39.84%, 37.35%, and 55.18% of the total effect of Hyperglycemia, HPV, and their combined impact on cervical cancer (all P < 0.05, **Figure 3**). Model adjusted for age, pregnancy, parity, total cholesterol, triglyceride, High -density lipoprotein cholesterol, Low density lipoprotein cholesterol, Albumin, Alanine aminotransferase, γ-glutamyl transpeptidase, total bilirubin, Specific protein. Dysglycemia and HPV similarly demonstrate a significant indirect effect mediated by Ki-67 levels, with an estimate of 0.08 (95% CI: 0.06-0.09, p<0.001). This suggests that abnormal blood glucose elevation and HPV contribute to cervical carcinogenesis, at least partially, by inducing Ki-67 overexpression and aberrant proliferation. Beyond this Ki-67-mediated pathway, dysglycemia exerts a smaller but still significant direct effect of 0.13 (95% CI: 0.011- 0.14, p<0.001) on cervical cancer risk.

These findings indicate that both HPV and dysglycemia promote cervical cancer development through common mechanisms involving Ki-67 upregulation and increased cellular proliferation, as evidenced by the significant indirect effects. However, each factor also appears to impact carcinogenesis through other direct pathways independent of Ki-67. This analysis provides evidence that Ki-67 overexpression represents a key mechanistic link between HPV, dysglycemia, and cervical cancer development. Nevertheless, additional Ki-67-independent pathways also contribute. Targeting proliferative signaling and improving metabolic health may help disrupt the oncogenic effects of these risk factors and reduce the burden of cervical cancer. Understanding these mediators is crucial for developing effective prevention and therapeutic strategies.

## 4 Discussion

The present study sheds light on the intricate relationship between HPV infection, dysglycemia, and cervical cancer development, emphasizing the mediating role of Ki-67. HPV infection significantly escalates cervical cancer risk, with an odds ratio surging to 152.85 (95% CI: 48.56–481.09). Consistent with our findings, research by Bruni et al. (2016) and Tan et al. (2018) underscores the significant association between HPV infection and cervical lesion risk. Bruni et al.’s global summary report underscores the relevance of HPV in cervical cancer, while Tan et al.’s study in Malaysian women focuses on HPV prevalence and type distribution (27, 28). Dysglycemia, encompassing both diabetes and prediabetes, emerges as a significant risk factor, with diabetes showing a particularly elevated odds ratio of 3.67 (95% CI: 2.41-5.6) compared to normoglycemic individuals. Similarly, prediabetes significantly increases the risk, with an odds ratio of 2.47 (95% CI: 1.75-3.47). Meanwhile, HPV infection, particularly with high-risk types such as HPV 16/18, substantially increases the risk of cervical cancer. Crucially, the study unveils a synergistic interaction between dysglycemia and HPV infection, amplifying cervical cancer risk beyond their individual effects. Among HPV-positive women, the presence of dysglycemia increases the odds of cervical cancer by over 3-fold compared to HPV-positive individuals with normal glycemic status. This synergy is further supported by measures of additive and multiplicative interaction. The attributable proportion (AP) was 0.71, indicating that approximately 71% of cervical cancer cases among individuals exposed to both dysglycemia and HPV infection may be attributable to their interaction, although again, caution is warranted given the non-significant RERI. Additionally, the synergy index (SI) was 3.45 (95% CI: 2.61–4.56, p < 0.001), providing statistically significant evidence for a multiplicative interaction. Some studies have also found that diabetes increases the risk of cervical cancer. For example, a Danish cohort study of 2,508,321 women found a higher incidence of cervical cancer among those with diabetes (IRR:1.13, 95% CI: 1.00-1.28) (29).Additionally, a meta-analysis of 19 studies reported a relative risk of 1.34 (95% CI: 1.10-1.63) for cervical cancer in women with diabetes (30). Furthermore, a study of 397,783 adults demonstrated a 30% higher prevalence of cervical cancer in diabetic individuals, even after adjusting for confounders (p=0.0011) (31).

Although the risk is relatively low, these studies included the general population, while our focus is on exploring the tumor-promoting effect of hyperglycemia during HPV infection. Another study had similar findings to ours: a retrospective cohort study of 328,994 diabetic individuals noted an increased cervical cancer risk in newly diagnosed type 2 diabetes cases (HR:3.46, 95% CI: 1.10-10.86, p=0.03) (12). However, these studies only found an association between dysglycemia and increased risk of HPV-related cervical cancer, but did not analyze the interaction. Our interaction analysis further confirmed the tumor-promoting effect of dysglycemia on HPV-related cervical cancer. While further research is needed to elucidate the mechanisms, these findings collectively suggest a potential impact of diabetes on cervical cancer risk.

Ki-67 is closely associated with cell proliferation and metastasis, playing a pivotal role in cervical cancer progression (32)and adverse outcomes. Studies consistently link Ki-67 overexpression with advanced cervical cancer stages, lymphatic metastasis, and poor prognosis (33–37). Moreover, both HPV infection and high blood glucose levels are correlated with elevated Ki-67 expression, suggesting a potential mediating role for Ki-67 in the pathways through which HPV and hyperglycemia promote cervical cancer development. Ki-67’s crucial role in cervical cancer progression aligns with our findings. Similar synergistic effects have also been observed in studies investigating the impact of diabetes on the risk of other cancers, where diabetes increases the risk of many cancers (38–42). Additionally, other studies have found that Diabetes Mellitus promotes Ki-67 expression and cell proliferation (26, 43). Our study found a similar effect, and we further analyzed the mediating role of Ki-67 in the increased risk of cervical cancer associated with HPV infection and dysglycemia. In this study, robust mediation analysis revealed Ki-67+ plays a significant role in the collective impact of Hyperglycemia, HPV, and their co-infection on cervical abnormalities. Specifically, Ki-67+ contributed to 39.84%, 37.35%, and 55.18% of the overall effect of Hyperglycemia, HPV, and their combined influence on cervical cancer, respectively.

While our study provides valuable insights into the complex interplay between HPV infection, dysglycemia, Ki-67 expression, and cervical cancer risk, several limitations should be acknowledged. Firstly, the cross-sectional design of our study limits our ability to establish causal relationships between the variables examined. Longitudinal studies with a prospective design would offer a more robust approach to elucidate the temporal sequence of events and infer causality. Secondly, while our analysis controlled for several confounding factors, residual confounding remains a possibility. Factors such as dietary habits, medication use, and comorbidities not accounted for in our analysis may influence the observed associations. Additionally, our study focused primarily on Ki-67 expression as a marker of proliferation, overlooking other potential molecular mechanisms involved in cervical carcinogenesis. Future research incorporating a broader array of biomarkers and molecular pathways would provide a more comprehensive understanding of the underlying mechanisms. Furthermore, our study population consisted primarily of individuals from a single geographic region, which may limit the generalizability of our findings to other populations with different demographic and socioeconomic characteristics. Moreover, the study’s sample size may have limited statistical power, particularly for subgroup analyses and interaction analyses. Larger sample sizes would allow for more robust assessments of effect modification and subgroup differences. Lastly, the lack of information on HPV genotypes beyond HPV 16/18 and detailed glycemic control measures, precluded a more nuanced analysis of these variables’ impact on cervical cancer risk. Despite these limitations, our study contributes valuable insights into the complex etiology of cervical cancer and underscores the need for further research to address these limitations and confirm our findings in diverse populations using longitudinal designs and comprehensive molecular analyses.

## 5 Conclusion

Dysglycemia and HPV infection synergistically elevate cervical cancer risk, providing compelling evidence of their independent and synergistic effects, likely mediated by Ki-67. These findings highlight the necessity for comprehensive screening and management of both HPV and dysglycemia in cervical cancer prevention. The synergistic interaction between these two factors significantly amplifies the risk of developing cervical cancer, surpassing their individual impacts. The Ki-67 marker appears to play a crucial role in mediating this synergistic effect, accounting for a substantial proportion of the combined impact on cervical cancer development. Further research is needed to elucidate the underlying molecular mechanisms and validate these findings across diverse populations and ethnic groups. Ultimately, targeting Ki-67-mediated pathways and exploring novel therapeutic interventions that modulate these pathways may offer promising avenues to mitigate the elevated cervical cancer risk observed in individuals with concurrent HPV infection and dysglycemic conditions such as prediabetes or diabetes.

## Data Availability

The raw data supporting the conclusions of this article will be made available by the authors, without undue reservation.
